# A Review of the Current Knowledge of Thermal Stability of Anthocyanins and Approaches to Their Stabilization to Heat

**DOI:** 10.3390/antiox10091337

**Published:** 2021-08-24

**Authors:** Simona Oancea

**Affiliations:** Department of Agricultural Sciences and Food Engineering, “Lucian Blaga” University of Sibiu, 7–9 Dr. Ion Ratiu Street, 550024 Sibiu, Romania; simona.oancea@ulbsibiu.com

**Keywords:** anthocyanins, heat stability, degradation kinetics, stabilization techniques

## Abstract

Anthocyanins are colored valuable biocompounds, of which extraction increases globally, although functional applications are restrained by their limited environmental stability. Temperature is a critical parameter of food industrial processing that impacts on the food matrix, particularly affecting heat-sensitive compounds such as anthocyanins. Due to the notable scientific progress in the field of thermal stability of anthocyanins, an analytical and synthetic integration of published data is required. This review focuses on the molecular mechanisms and the kinetic parameters of anthocyanin degradation during heating, both in extracts and real food matrices. Several kinetic models (Arrhenius, Eyring, Ball) of anthocyanin degradation were studied. Crude extracts deliver more thermally stable anthocyanins than purified ones. A different anthocyanin behavior pattern within real food products subjected to thermal processing has been observed due to interactions with some nutrients (proteins, polysaccharides). The most recent studies on the stabilization of anthocyanins by linkages to other molecules using classical and innovative methods are summarized. Ensuring appropriate thermal conditions for processing anthocyanin-rich food will allow a rational design for the future development of stable functional products, which retain these bioactive molecules and their functionalities to a great extent.

## 1. Introduction

The nutritional community-based interventions are related to programs aimed not only at correcting nutritional deficiencies but also at changing diet in order to positively impact the course of an illness. Therefore, a diet rich in antioxidant molecules or based on functional foods has gained great popularity among public health programs because of the reduction of cancer risk or other chronic diseases known as leading causes of death. Beyond their nutritional role, the constituents of a food matrix play a protective role against many human diseases, advancing the perception of food as functional and nutraceutical [1]. The new direction of an interdisciplinary approach in conducting food research (chemistry of natural products, food sciences, clinical nutrition) creates opportunities for more detailed data on food chemical composition, effects of industrial processing on their components and clinical data validating the mechanism of action and bioavailability of the bioactive compounds [2]. Food plants have been traditionally considered the richest source of health-promoting compounds resulting from their metabolism, either primary or secondary. Secondary metabolites are non-nutrient compounds, classified into three main classes, isoprenoids, phenylpropanoids and alkaloids, whose representatives are constantly researched and updated in scientific databases [3]. Anthocyanins are natural compounds of the phenylpropanoid class responsible for the red, blue, or purple color of fruits, flowers, or leaves, being largely distributed in plant cell vacuoles. Based on their physical chemical and biological properties, such molecules easily found applications in nutrition (dietary antioxidants), medicine (therapeutic agents), or industry (food, textile—natural colorants, preservatives and ingredients, photoelectrochemical cells) [4]. The most important issue for which these compounds cannot compete with synthetic additives is their low stability, in particular under high pH and temperature. However, compared to other natural colorants (betalains, carotenoids, chlorophylls), anthocyanins are more stable in relation to heat [5], which calls for further applied research in this direction. 

Academic interest in researching and using anthocyanins goes beyond their coloring capacity, being directed towards their beneficial validated health effects, such as antioxidant, anti-inflammatory, anti-obesity, anti-diabetes, anti-tumor, anti-ulcer, and neuroprotective [4]. 

The main structure of the anthocyanin molecule consists of a 2-phenylbenzopyrylium (flavylium) heterocyclic C–15 skeleton (called aglycon or anthocyanidine) containing –OH or –OCH_3_ groups, bearing one or more sugar or acylated sugar residues. Studies on structure–activity relationship have shown that the presence of –OH groups increases blue color and reduces stability, while that of –OCH_3_ groups increases redness and stability [5]. Stability is also higher if acylation of the sugar moiety with aromatic or aliphatic acids occurs. 

The nutritional intervention for correcting the oxidative damage of human cells has recently been considered as a key factor for preventing the risk of chronic degenerative diseases [6]. The oxidative damage is the consequence of the action of reactive oxygen species (ROS) so-called “free radicals”, such as superoxide radical anion (O_2_^•−^), hydroxyl radical (HO^•^), hydrogen peroxide (H_2_O_2_) and singlet oxygen (^1^O_2_). These chemical species produce in vivo lipid peroxidation in cell membranes, inactivation of enzymes, activation of specific signal pathways, toxic products, and DNA damage. Oxidative stress is the consequence of an impaired balance between oxidative conditions and antioxidant mechanism. Among different dietary antioxidants, anthocyanins may prevent or inhibit oxidative stress by scavenging free radicals [7] or by other mechanisms, such as metal chelation and oxidative enzyme inhibition [8]. During oxidation, anthocyanin molecules act by donating H atoms or by single electron transfer, as function of the ring orientation and the number of hydroxyl groups. Tena et al. [7] reported that the antioxidant activity of anthocyanidins (delphinidin, cyanidin, pelargonidin) is higher than that of the corresponding anthocyanins, probably due to their increased chemical reactivity. Acylation of anthocyanins increases their antioxidant activity, while glycosylation decreases it. Different methods used for measuring the antioxidant activity have been described, such as FRAP (Ferric Reducing Antioxidant Power Assay), ABTS (2,2′-Azino-*bis*(3-ehtylbenzothiazoline-6-sulfonic acid) Diamonium Salt Assay) measuring TEAC (Trolox Equivalent Antioxidant Capacity), CUPRAC (Cupric Ion Reducing Antioxidant Capacity Assay), DPPH (Diphenyl-1-Picrylhydrazyl Assay), ORAC (Oxygen Radical Absorbance Capacity), each type influencing the mechanism of the antioxidant activity of particular anthocyanins. As Tena et al. pointed out [7], the correct evaluation of anthocyanin antioxidant activity should be done from a multiparametric viewpoint: source, total concentration, chemical structure, pH and mechanism of reaction. Anthocyanins become unstable under different environmental conditions, mainly pH, temperature, light, and oxygen, factors which are critical for the effective control of their extraction, processing or storage. All three practices may involve the use of heat. Degradation of anthocyanin pigments is associated with their color fading. Most studies investigated the pH influence on stability, confirming that anthocyanins are stable at low pH values (<3), which may limit their use to high acidic food products, such as fruit juices and certain dairy products (yogurt, kefir, some cheeses). The investigation becomes more complex when thermal stability of anthocyanins is studied, due to the multitude of generated degradation products, varying according to the plant source, the environmental conditions, and the proposed study design. Sui et al. [9] observed that increasing temperature has a greater negative impact on the stability of cyanidin-3-*O*-glucoside and cyanidin-3-*O*-rutinoside from black rice than increasing pH from 2.0 to 6.0.

Because lots of plant foods are thermally processed before consumption, in order to inhibit microbial growth, remove water, or inactivate enzymes, heating will impact on the anthocyanin content, being a major concern in particular for juice and jam production. Thermal stability experiments were usually performed by storage of anthocyanin extracts or solutions of pure individual compounds at different temperatures under isothermal mode for various heating times, after which the content of residual total or individual anthocyanins was measured. The obtained experimental data can be used to determine the kinetic parameters of the degradation, mainly employing the Arrhenius model. Other models, such as the thermodynamic Eyring model, which evaluates the enthalpy and entropy of activation, and the Ball model, an approach used for studying the microbial food deterioration based on the decimal reduction time related to temperature, have also been explored [10]. In order to minimize the degradation of anthocyanins during thermal processing of anthocyanin-containing foods, a well formulation of the thermal conditions is required so that the product quality is not affected. Because the antioxidant activity of anthocyanins also depends on their total content, a well-conducted thermal processing will additionally preserve their antioxidant properties, or even improve them due to the generation of other antioxidant products during heat-induced reactions [11,12]. 

The purpose of this study was to give in-depth updated knowledge on the molecular mechanism and kinetics of anthocyanin degradation during heating, to comparatively describe the thermal behavior of anthocyanins both within natural extracts (models) and in real food products, which could be considerably different, by presenting some practical useful examples. In the final part, the review describes both common and innovative methods tested to enhance heat stability of anthocyanins, based on up-to-date information. A literature review in this field is in great demand, in particular for researchers involved in anthocyanin extraction and application and for food manufacturers mainly interested in developing novel products and functional food. Given the mountains of papers published on the general topic of anthocyanin stability, only recent papers related to anthocyanin heat and thermal stabilization strategies have been discussed hereby, contributing to the advancement of knowledge in this particular field. 

Several multidisciplinary databases (Scopus, Web of Science) and publisher databases (ScienceDirect, SpringerLink) were searched to identify full text original contemporary articles published on the subject of anthocyanin degradation and stabilization related only to heat. Most publications cited were found by using search terms: “anthocyanin degradation”, “anthocyanin degradation kinetics”, “anthocyanin thermal stability”, “anthocyanin heat resistance”, “anthocyanin stabilization”, etc. The relevance of the given list of publications was further assessed by examining the title and the abstract. Of the total number of papers reviewed, 76% were published in the last 10 years, of which 70% were published in the last 5 years.

## 2. Chemical Degradation of Anthocyanins during Heating: Molecular Mechanism and Kinetics

Anthocyanins, in particular when isolated from their native environment, are unstable and degrade to different extents during heating, in relation to several process parameters and the presence of other impacting molecules. Knowing the mechanism by which these biomolecules decompose is essential for maximizing their biologically active properties and visual quality when industrial thermal processing or domestic cooking is applied to anthocyanin-rich materials.

### 2.1. Chemical Reversible Transformations of Anthocyanins

**Figure 1:**
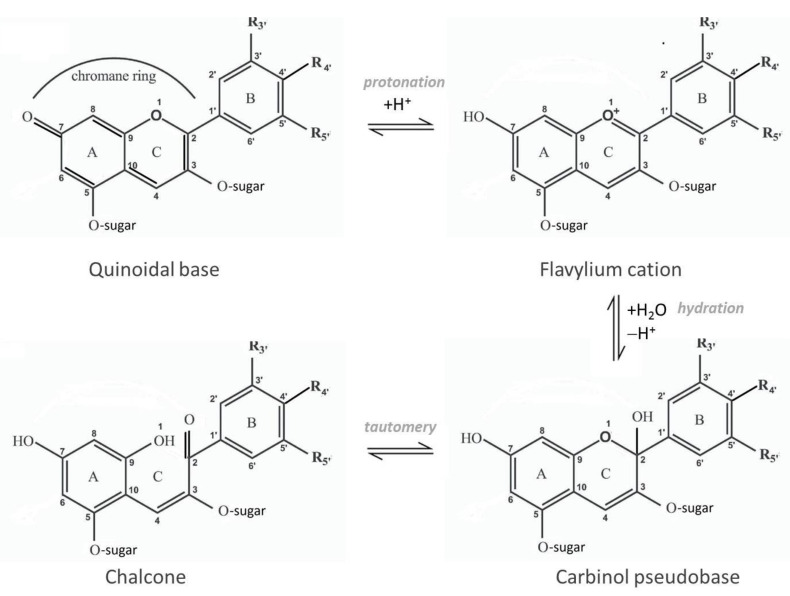
Structural reversible chemical transformations of anthocyanins.

Anthocyanins suffer reversible structural transformations under acidic aqueous media, with a final tautomeric reaction conducting to the open-form called chalcone, as reported by Brouillard and Delaporte in a highly cited paper [13]. Their obtained results brought forward the understanding of thermal degradation of anthocyanins. The reactions of the structural transformations presented in Figure 1 have been established as follows: (1) the intramolecular proton transfer “quinone-phenol” by which the quinoidal base is transformed into the colored flavylium cation; (2) the hydration of flavylium cation into the colorless carbinol pseudobase/hemiketal form; and (3) the water (solvent)–catalyzed tautomery by which the carbinol pseudobase is transformed into an aromatic ketone called chalcone, mainly the *cis*-chalcone isomer, through opening of the pyrylium ring C. The chalcone form prevails in anthocyanins lacking a substituent at position 3 (monoglycosides). The reported thermodynamic study of the carbinol-chalcone tautomeric equilibrium showed that the ring-opening reaction is always endothermic, so that increasing the temperature of anthocyanin solutions will favor the chalcone form over the other forms (quinoidal base, flavylium cation or carbinol pseudobase). Under acidic media (pH < 4), the flavylium cation and quinoidal base occurs, the first one being favored by very low pH values (<1), while under moderately acidic media (pH 4–6) and at room temperature, anthocyanins exist in both forms of the tautomeric reaction, carbinol and chalcone [14]. At higher pH values, anthocyanins are degraded to various compounds, to different extents.

### 2.2. Kinetics and Thermal Degradation Route of Anthocyanins

**Figure 2:**
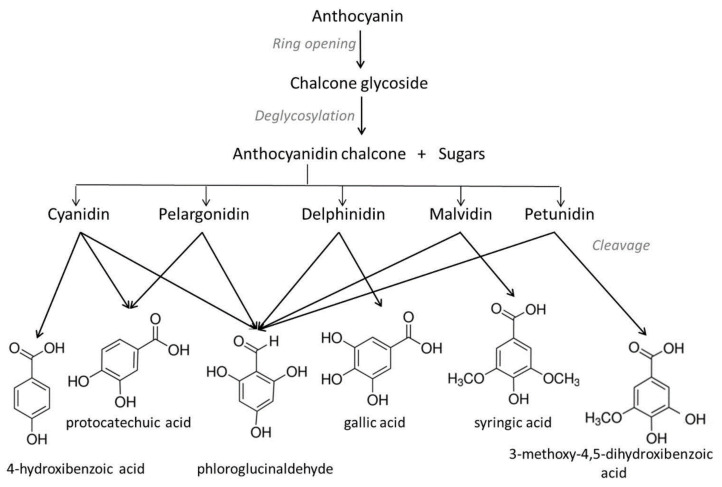
Possible mechanism of thermal degradation of the most common non-acylated anthocyanins.

The rate constant, *k*, represents a specific and constant characteristic of a chemical reaction, which depends on temperature and catalyst and not on concentration. Therefore, calculation of *k* allows the comparison of the rates of different reactions, performed under similar conditions (temperature, environment). According to the theoretical and experimental kinetic equations studied on malvidin 3-*O*-glucoside (monoglucoside) at 25 °C, the rate constants (*k*) of its structural transformations as presented in Figure 2, are as follows: *k* of protonation = 6.7 × 10^8^ s^−1^, *k* of deprotonation = 4.7 × 10^4^ s^−1^, *k* of hydration = 8.5 × 10^−2^ s^−1^, *k* of dehydration of the carbinol = 34 s^−1^, *k* of pyrylium ring-opening = 4.5 × 10^−5^ s^−1^ and *k* of cyclization = 3.8 × 10^−4^ s^−1^ [13]. It has been demonstrated that the presence of a glucose residue at position 5 (ring A) has a minimal influence on the kinetics of the proton transfer equilibrium, but significantly increases the hydration rate, slowing down the reverse reaction [13].

Similarly, other authors suggested that the thermal degradation of anthocyanins, in particular at pH 3.5, follows the pathway of intermediate chalcone and final derivatives of aldehyde and benzoic acid, structurally differentiated based on the corresponding anthocyanidin [15,16,17,18,19], as illustrated in Figure 2 for the most abundantly distributed anthocyanidins in fruits and vegetables (cyanidin, pelargonidin, delphinidin, malvidin and petunidin). Combined hydrolytic and autooxidative reactions are probably involved [20]. Phloroglucinaldehyde results from the C3–C4 cleavage, while phenolic acids such as 4-hydroxibenzoic, protocatechuic, gallic and syringic are generated through the cleavage of C2–C3 bonds.

In addition to the protocatechuic acid and phloroglucinaldehyde products, the thermal oxidative degradation of cyanidin-3-*O*-[Glc-2-*O*-Glc]-5-*O*-Glc, cyanidin-3-*O*-[(6-*O*-*p*-coumaroyl)-Glc-2-*O*-Glc]-5-*O*-Glc, and cyanidin-3-*O*-[(6-*O*-*p*-coumaroyl)-Glc-2-*O*-(2-*O*-sinapoyl)-Glc]-5-*O*-Glc, can also conduct to other compounds, e.g., 3,5,7-trihydroxycoumarin and 2,4,6-trihydroxyphenylacetic acid as reported in Fenger et al. [21]. Recently, some researchers showed that gallic acid and phloroglucinaldehyde resulting from eggplant anthocyanin degradation are further oxidized into pyrogallol and phloroglucinol, respectively [22].

Obviously, thermal degradation of anthocyanins occurs to different extents, depending on the raw starting material, pH and co-pigments. It has been shown that some anthocyanins, in particular monoglycosides, e.g., cyanidin-3-*O-* glucoside and pelargonidin-3-*O*-glucoside from blackberries and strawberries, are more susceptible to heat [23]. Acylation and methoxylation usually enhance the stability of anthocyanins to heating [23]. The glycosyl groups of many anthocyanins are acylated with organic acids, generating the so-called “acylated anthocyanins”. Glycosyl acylation enhances the chemical stability of anthocyanins under different physicochemical conditions, such as slightly acidic and neutral media, light and heat, due to different mechanisms: (*1*) the decrease in polarity and creation of a steric hindrance by acyl groups, effects by which the nucleophilic attack of water on the flavylium ion is lowered; (*2*) the intramolecular co-pigmentation caused by aromatic acyl groups; (*3*) formation of zwitterions in case of acylation with dicarboxylic acids, the remaining –COOH group being dissociated, thus generating protons that decrease the pH and favor the flavylium cation [24]. The results of the study of Fenger et al. [21] on non-acylated and acylated red cabbage anthocyanins, showed that at pH 7.0 and 50 °C, the blue color stability of diacylated anthocyanin is significantly higher than that of its non- and monoacylated counterparts. In the above study, the color loss of anthocyanins diacylated with sinapoyl and *p*-coumaroyl residues, during heating, is due to: (a) non-oxidative degradation by deacylation (hydrolysis) and intramolecular acyl transfer (migration of acyl group, trans-esterification), more prominent for sinapoyl, and (b) irreversible oxidative alteration to different products. These findings are essential for technological applications, as acylation of anthocyanins significantly prolongs their half-life compared to non-acylated anthocyanins, particularly in vegetable juices or colorants. While non-acylated anthocyanins are 99% colorless at pH 7.0, the monoacylated and diacylated forms are 15% and 80%, respectively, colored at neutral conditions [21]. However, under heating at 95 °C, acylated anthocyanins from black carrot firstly decompose to the corresponding anthocyanidin and acyl-glycoside, the latter suffering further deglycosylation and formation of phenolic acids (coumaric, ferulic, sinapic) [11].

The kinetic reaction approach has been largely employed to study the quality of food issues and to predict the impact of processing on critical parameters of food quality. Chemical kinetics give useful information about the mechanism of the conversion of reactants into products. Because it is impossible to measure the reaction rate itself during a kinetic experiment, the content of the target compound is measured as a function of time. By studying the time evolution of processes (e.g., anthocyanin degradation), kinetic parameters such as reaction order (n), rate constant (*k*), half-time (*t*_1/2_) and activation energy (*E_a_*) can be calculated.

Temperature is an important environmental factor that greatly influences the rate of reactions that occur in food. Degradation of anthocyanins during isothermal heating follows a first-order kinetic model, with a good regression coefficient R^2^, as reported in most studies [16]. However, due to non-isothermal conditions encountered when heating semisolid or solid food, a kinetic modeling is required by using nonlinear regression techniques [25].

It is well established that anthocyanins are more stable at very low pH values (pH = 1). Higher values (pH > 3.0) are more typically found in major plant food processing (juices), so that thermal stability studies performed at slightly acidic or even neutral pH will be of relevant practical interest.

One key parameter of the thermal anthocyanin degradation kinetics is the half-time, *t*_1/2_—the time required for the consumption of 50% of the initial anthocyanin concentration, being calculated using the following equations:

(1) $$ln\frac{C}{C_{0}}=-kt$$

(2) $$t_{1/2}=\frac{ln2}{k}$$

where *C* is the anthocyanin content at time *t*, *C*_0_ is the initial anthocyanin content. 

Different kinetic models (Arrhenius, Eyring, Ball) may be used to estimate the anthocyanin losses during industrial food processes. The Arrhenius model, derived from the thermodynamic laws, relates the reaction rate constant (*k*) to temperature [26], according to the following equation:

(3) $$k=k_{A}\times e^{-\frac{E_{a}}{RT}}$$

where *k_A_* is the Arrhenius constant (frequency factor or pre-exponential factor, independent of temperature), *E_a_* is the activation energy: the minimum kinetic energy required for the molecules to react (J mol^−1^), R is the universal gas constant (8.314 J mol^−1^ K^−1^) and *T* is the absolute temperature (in Kelvin). 

By converting the above equation into the natural logarithmic form, the following relation is obtained:

(4) $$lnk=lnA-\frac{E_{a}}{RT}$$

A chemical reaction can be monitored at different temperatures by measuring the content of specific bioactive compounds in a time-dependent way. The Arrhenius plot of Equation (4), *lnk* vs. 1/*T*, allows the prediction of the reaction rate at a given temperature, and the calculation of *E_a_* from the slope of the line (*E_a_*/R): the lower the *E_a_*, the higher the reaction rate (anthocyanin degradation) and *viceversa*. Some reported activation energies (*E_a_*) of anthocyanin degradation during heating at temperatures up to 80, 90 or 100 °C of fruits intended for juice production, are as follow: 94 kJ/mol (blackcurrant), 92 kJ/mol (blueberry), 81 kJ/mol (Maqui), 68.042 kJ/mol (acerola), 66.04 or 55.81 kJ/mol (blood orange), 64.89 kJ/mol (grape), 58.95, 36.99 or 23.96 kJ/mol (blackberry), 42.8 kJ/mol (açaí), 21.6 kJ/mol (wild strawberry) [10,27,28,29,30,31,32]. Other values of *E_a_* and in addition of *k* and *t*_1/2_ are presented in Table 1 for a selection of food issues. Most of the published studies were accomplished at temperatures up to 100 °C, under moderately acidic or neutral media. 

Based on synthesis of data presented in Table 1, the mean values of anthocyanin half-life were 19.7 h for fruits and 22.28 h for vegetables, at 60 °C and low pH values (3.0–3.5), conditions at which most of the investigations were performed, and drastically decreased with temperature, e.g., to 7.76 h for fruits and 13.09 h for vegetables, at 80 °C. The results confirm that the anthocyanin degradation kinetics at a given temperature are influenced by monitoring time, experimental pH, plant cultivars and other environmental factors [16]. At higher pH values (5–6) the *t*_½_ decreased faster at 60 °C than at 80 °C, by 76% in blueberries and 86% in black rice, as can be deducted from Table 1. Pure cyanidin-3-*O*-glucoside, the major anthocyanin found in fruits and vegetables, showed a half-life of 6.4 h when heated at 80 °C for 2 h, at pH 3.0, value which decreased by 72% at pH 6.0, confirming that pH plays an important role in stabilizing anthocyanins under thermal treatment. Cyanidin-3-*O*-glucoside seems more sensitive to pH and temperature compared to other anthocyanins, e.g., cyanidin-3-*O*-rutinoside [45].

The Arrhenius model, the thermodynamic Eyring model and the Ball model were validated in a study on prediction of anthocyanin loss during heating the extract of roselle (*Hibiscus sabdariffa* L. cv. Vimto) giving similar results and showing estimated values close to the experimental ones, under isothermal and non-isothermal conditions (simulated pasteurization) [10].

## 3. Behavior of Anthocyanins within Extracts and Real Foods, as Main Influence of Temperature and Time

### 3.1. Anthocyanin Thermal Degradation in Crude and Purified Extracts

The influence of temperature on the degradation of anthocyanins can be studied in model solutions in a simple way by which interactions with other molecules are mostly avoided. Most scientific studies on thermal degradation of anthocyanins were conducted on their crude extracts (acidified or non-acidified alcoholic extracts), under heating temperatures lower than 80 °C. Little information is available on degradation kinetics of purified anthocyanins. The anthocyanin thermal stability in the two types of extracts, crude or purified, might be different depending on the presence of other compounds in the crude extracts (sugars, other phenols, organic acids, salts, etc.) which can enhance the anthocyanin stability. Thus, according to some published comparative studies on commercial anthocyanin extracts and açaí fruit extracts, purified anthocyanins degraded more rapidly compared to anthocyanins in the crude extracts, due to inter- and intramolecular co-pigmentation reactions [46,47]. Another study conducted on açaí fruits showed that the crude extracts of anthocyanins were 80 times more stable than the purified ones, at pH 2.2, and 24 times more stable at pH 3.0 [48]; however, the study did not investigate their thermal stability. The authors’ findings are of practical importance because purification usually involves high costs, and it is of biological relevance based on the synergistic action of co-occurring bioactive compounds, conducting to superior biological activities, particularly antioxidant capacity [49].

The anthocyanin loss due to heating of crude and purified extracts is presented in Table 2.

According to the findings summarized in Table 2, less than 20% of anthocyanins from fruits degraded at temperatures up to 60 °C or 70 °C under acidic conditions and low heating time (approx. 2 h), while lower percentages ~5–9% anthocyanins originating from vegetables degraded under similar conditions. Instead, an aqueous anthocyanin extract from butterfly pea blue petals presented good thermal stability at 60 °C and 70 °C and pH 3.6 and 5.4, lasting for 360 min and good color retention (90%) at higher temperatures such as 80 °C [54]. However, usually the time required for heat treatment during processing of fruits and vegetables in order to control microbial hazard is in the range of minutes at temperatures of 70 °C or 90 °C [56].

The content of total anthocyanins significantly reduces during heating under neutral and alkaline pH. 

Non-acylated anthocyanins are found in fruits (elderberries, blackcurrants, blackberries, blueberries, grapes), while mono-, di-, tri- or tetra-acylated anthocyanins are distributed in vegetables (purple and black carrot, red radish, red cabbage, purple sweet potato, purple corn) and flowers (*Matthiola longipetala*, *Iberis umbellata*, *Ionopsidium acaule*, *Rhoe spathacea*, *Clitoria ternatea*, *Gynura bicolor*, *Ajuga reptans*) [57]. Acylated anthocyanins are more stable in aqueous solutions, due to intramolecular co-pigmentation and generation of an acidic environment [57]. Similarly, Zozio et al. [55] showed that acylated anthocyanins from black carrot were more stable to temperature change in the range of 20–50 °C than non-acylated anthocyanins from Andean blackberry or açai fruits. 

Highly different results on thermal degradation of anthocyanins from vegetables have been reported with red cabbage extracts, based on the type of extraction. Thus, acidified ethanol extracts (pH 5.0) showed 6.5% anthocyanin degradation after 2 h of heating at 70 °C and 33.33% at 90 °C, respectively, according to Ekici et al. [51], while aqueous extracts (pH 5.5) showed ~30% anthocyanin degradation at 70 °C and ~54% at 90 °C after 2 h according to Fernández-López et al. [52]. The method of obtaining anthocyanin extracts and the design approach for the thermal degradation studies (based on absorbance decay or total content of anthocyanins) clearly have a strong influence on the results, in addition to other described features.

In a study reporting the stability of individual anthocyanins identified in bilberry methanolic extract heated to 80 °C, 100 °C, or 125 °C, the authors found no statistically significant differences among ten anthocyanin samples, but a tendency of cyanidin-arabinoside, delphinidin-arabinoside and malvidin-arabinoside to be more heat sensitive than their corresponding glucosides or galactosides [58].

### 3.2. Behavior of Anthocyanins during Processing Involving Thermal Treatment of Food Rich or Enriched with Anthocyanins

Several food industrial processes (blanching, pasteurization, sterilization, incubation, evaporation, steaming, drying, cooking, baking) involve high temperatures, >60 °C, for a certain period of time, applied to raw or purified sources of anthocyanins. Thermal treatment is required in food industry mainly to extend food shelf-life and safety, but also to improve nutrient levels or to produce convenient food for home consumption, out-of-season, or novel food products. Temperature is a critical parameter in food processing, but at the same time it is the major factor that affects the anthocyanin content of the final product, in strong relation to magnitude and duration of heating, and interactions with other molecules that induce various chemical changes.

The effects of different heat treatments on the anthocyanin availability in foods rich in such biomolecules are summarized in Table 3.

Generally, thermal processing of simple food matrices, fruits, and vegetables, appears to highly impact on anthocyanins, with their content loss varying from 28 to 80%, as concluded from studies cited in Table 3. In relation to the high activation energy values, the influence of temperature on anthocyanin degradation in real heating situations becomes considerable only for high residence time, as shown by a reported kinetic and thermodynamic study on acerola (*Malpighia emarginata* L.) pulp [28]. According to that study, the anthocyanin loss in a simulated industrial pasteurization tubular system was <1% at different temperatures (60, 70, 80 and 90 °C) for 20 s.

Thermal behavior of anthocyanins could be different within more complex food matrices, e.g., in anthocyanin-enriched/fortified foods, not following the general consensus that heating greatly decreases anthocyanin stability. However, it is difficult to distinguish the effects of heating from the effects of food matrix [72]. Some researchers reported that the time of heating seems to influence to a greater degree the loss of anthocyanins than the temperature, as confirmed in particular foods such as bakery products [73]. 

Non-isothermal kinetic modeling of anthocyanin degradation during baking of bread prepared from flour fortified with anthocyanin-rich black rice powder at three different temperatures, 200 °C, 220 °C, and 240 °C for different times (2–12 min) showed lower *k_ref_* values, particularly in the crumb, than other reported values for aqueous systems, e.g., fruit juices heated to 120–140 °C [74]. The kinetic parameters for the degradation of anthocyanins (cyanidin-3-*O*-glucoside and cyanidin-3-*O*-rutinoside) were determined by the Arrhenius model using the equation:

(5) $${k=k}_{ref}{\times e}^{-\frac{E_{a}}{R}(\frac{1}{T}-\frac{1}{T_{ref}})}$$

where *k_ref_* is the rate constant at the reference temperature (*T_ref_*) of 125 and 65 °C for crust and crumb samples, respectively (the average values of the temperature range they experienced during baking). Other studies suggested interactions of anthocyanins with flour proteins and polysaccharides other than gluten and starch, which stabilize anthocyanins in complex matrices such as bread [75]. The authors of the above-mentioned published paper explained the higher anthocyanin thermal stability in bread by the low oxygen availability, which reduces the oxidative reactions. Some bakery products (bun, breadstick, and biscuit) enriched with red grape skin extract retained significant amounts of anthocyanins (total anthocyanins and malvidin-3-*O*-glucoside) after baking, as follows: 95.9% in the bun, 98.6% in the biscuit, and 63.28% in the breadstick [76]. Additionally, new chemical compounds (new acylated anthocyanins) were detected in the final product, probably as a result of the reactions occurring during fermentation in bun production [76]. A similar study on biscuit dough fortified with anthocyanins and baked at 160 °C for 10 min showed a two-fold decrease of anthocyanin degradation rate constant compared to control aqueous system, and three fold decrease when compared to a model blackberry juice, suggesting the great influence of matrix effects [77]. The calculated activation energy was *E_a_* = 87 kJ/mol. The study conducted by Zhang et al. [78] on an eggplant anthocyanin fortified model food system (cookies), which suffered steaming and boiling, showed lower *k* values (0.24 and 0.38 h^−1^) and higher *t*_1/2_ (2.86 and 1.79 h) for steamed and boiled cookies, respectively, compared to control anthocyanin samples subjected to the same heat treatment (0.71 and 0.39 h^−1^; 0.98 and 1.78 h).

The degradation of anthocyanins in blue corn-based extruded nixtamalized products followed a first-order model at temperatures ranging from 60 °C to 90 °C at pH 2.5, with the determined thermodynamic parameters showing that the process was endothermic and non-spontaneous [79]. The authors of the paper showed that anthocyanin degradation kinetics of extruded corn flour and of prepared tortilla were similar, although tortilla making involved much higher temperatures (300 °C) than those applied in the extrusion process, probably in relation to the matrix protective effects, which caused a slower heat flow to the center of the tortilla due to some anatomical parts of the kernel.

A study on anthocyanin enriched egg products (pancake, omelet) that have undergone thermal treatments (2.5 min at 250 °C and 2 min at 270 °C for pancake; 2 min at 250 °C and 45 s at 250 °C for omelet) showed a higher anthocyanin recovery in pancake (74.5%) than in omelet (31.4%) [80]. Moreover, the study indicated that the inclusion of anthocyanins in food matrices, particularly into solid ones, contributed to a lower intestinal degradation.

The strong antioxidant potential of anthocyanins makes them valuable for replacing synthetic additives in meat and for developing various functional foods [81,82,83,84]. However, anthocyanins added to meat become unstable and less bioavailable under thermal treatment conditions. Little information is given about the fate of anthocyanins in meat products subjected to heating, while most investigation focused on the shelf-life of raw functional meat products during storage [85]. Among several explored strategies to improve mulberry anthocyanin retention in hot processed minced pork slices, a procedure of drying at 40 °C for 10 h followed by baking at 150 °C for 3 min was the best method for acquiring maximum retention of anthocyanins, 59.62% [86]. Another study showed that Chinese-style sausages 0.3% enriched with roselle (*Hibiscus sabdariffa* L.) extract and processed by drying at 60 °C for 24 h retained their color well, with values similar to those of control samples [87]. 

## 4. Exploring Methods Designed to Enhance the Stability of Anthocyanins to Heat

The use of anthocyanin-based products in industrial applications (food, pharmaceutical, cosmetic and textile) is limited by their stability, in particular to heating and pH, such molecules being thermally stable at low pH. In order to prove their biologically active properties and the resulting health benefits, anthocyanins need first to withstand food processing. Consequently, a strategy for thermal stabilization is strongly required, in particular at pH >3.5 and neutral conditions, so that innovation in such technologies and approaches are encouraged. For a detailed classification of available techniques for enhancing stability of anthocyanins for food applications, readers are also invited to study the information updated until 2017, published by Cortez et al. [88]. The present section reviews up-to-date studies on advanced techniques for anthocyanin stabilization to heat.

The majority of research has been performed to investigate the effects of various molecules added to natural extracts in order to improve the thermal stability of anthocyanins, sometimes in high ratios which are impractical for technological application.

Simple sugars like glucose and trehalose added in a concentration of 10% to blackberry juice (heated at 90 °C) determined an increased stability of anthocyanins during storage, particularly in the presence of a co-pigment (chlorogenic acid) [89]. 

Anthocyanins, in the form of flavylium cation, bind different metal ions through a reaction which prevents the formation of the colorless carbinol pseudobase, conducting to enhanced heat stability [20,90]. An aqueous solution of cyanidin-3-*O*-glucoside demonstrated good stability to heat (60 °C, 80 min) under weakly acidic conditions (pH = 6.0) by the addition of Fe^3+^, particularly in combination with anionic polysaccharides (alginate, carrageenan, pectin), which inhibited complex aggregation, as shown by Tachabana et al. [91]. Interestingly, when Fe^3+^ was replaced by Fe^2+^ an improvement of stability was found, but this decreased when alginate was further added, probably due to a stronger interaction of Fe^2+^ with alginate that disturb the complex of anthocyanin–Fe^2+^.

Anthocyanin thermal stability was improved in case of butterfly pea (*Clitoria ternatea* L.) extract mixed with catechin at a ratio of co-pigment/anthocyanin 100/1 heated at 90 °C up to 60 min and pH 3.5, resulting in a ~2-fold decrease of the degradation rate constant (*k*) and increase of half-life of these antioxidant molecules [92]. Co-pigmentation of anthocyanins with other molecules which may even be parts of food components is one of the most frequently used methods for their stabilization through associations, including resistance to heat. Intermolecular co-pigmentation with various phenolic compounds such as flavonoids, protocatechuic, *p*-hydroxybenzoic, vanillic, syringic, gallic and ferulic acids, rutin, and catechin has been described [88]. The possible mechanisms of action are based on H-bonding, hydrophobic/ionic interactions, or intermolecular stacking [20,88]. Stabilization of anthocyanins from açaí fruits using tannic acid indicated a significant half-life increase of anthocyanins in purified extracts compared to that of the crude extracts [48]. 

Micro-encapsulation of anthocyanins using biopolymers (proteins, polysaccharides) does not seem to be highly influenced by pH and leads to binding of flavylium cation or hemiketal form to the biopolymer through weak interactions (H-bonds, van der Waals forces) [20,88]. Such techniques proved efficient at stabilizing anthocyanins subjected to heat treatments [93,94,95]. Encapsulation of anthocyanins from jabuticaba (Brazilian grapetree) either with calcium-alginate or polyethyleneglycol using supercritical CO_2_ led to an increased stability to light and temperature [5]. Micro-encapsulation of juçara fruit anthocyanins by spray/freeze drying with maltodextrin and arabic gum indicated that the thermal stability of freeze dried samples improved most significantly at a ratio of fruit pulp and polymeric matrix of 2:3, as measured by thermogravimetric analysis (TGA) and DSC assays [96]. Micro-encapsulation of *Hibiscus sabdariffa* L. calyces aqueous extract with whey protein isolate and/or polydextrose by freeze-drying generated stable powders up to 210 °C, as shown by TGA analysis [93]. The authors also conducted an accelerated stability test at 40 °C and 60 °C, for 28 days, using differently prepared powders, their results indicating a better retention of total anthocyanins up to 53% in samples prepared only with polydextrose by freeze-drying, under conditions of lower temperature (40 °C) and lower relative humidity (75%). Encapsulation of blackberry anthocyanins (cyanidin-3-*O*-glucoside) using β-cyclodextrin showed a decrease of the degradation rate constant, *k* [97]. Similarly, the thermal stability of the anthocyanin extract of *Kadsura coccinea*, a valuable Chinese medicinal plant, as measured by TGA assay was improved through complexation with β-cyclodextrin or its derivative (2-hydroxypropyl-β-cyclodextrin), showing less weight loss before 290 °C [98].

Nanoliposomes of anthocyanins primarily containing cyanidin-3-*O*-glucoside and peonidin-3-*O*-glucoside, formed with lecithin and cholesterol at a ratio of 5.98 showed an anthocyanin retention rate of 85.60% during storage at 25 °C for 16 days [99]. The effects of pH on the stability of the anthocyanin nanoliposomes indicated an increased retention of encapsulated anthocyanins at lower values (pH = 3.0) compared to that under neutral condition (pH = 7.0).

Another technique for anthocyanin stabilization was explored by mixing the anthocyanin colorant ColorFruit^®^ Violet 100 WS with yeast mannoproteins (~10% proteins and 90% carbohydrates) at pasteurization and sterilization temperatures, under neutral conditions (pH = 7.0) [100]. The complex formed by hydrophobic interactions showed 4 to 5-fold increase of half-life of anthocyanins and maintenance of their antioxidant activity.

Stabilization of anthocyanins extracted from different fruits (grape, blackberry, blackcurrant, cranberry) and vegetables (black carrot, red cabbage) with compounds containing thiol groups (cysteine, glutathione, dihydrolipoic acid) was found to protect them from degradation at pH = 7.0 and 37 °C [88]. 

Anthocyanin dispersions with co-polymers, such as those derived from the Maillard reaction (whey protein isolate glycated with glucose) showed good stability of cyanidin-3-*O*-glucoside to heating at 80 °C up to 80 min and improved antioxidant activity [44]. The complexes were formed through hydrophobic interactions as confirmed by fluorescence spectroscopy. Another study similarly reported an enhancement of anthocyanin (cyanidin-3-*O*-glucoside) stability under heat treatment at pH 3.0 and 6.8 in association with silkworm protein-glucose conjugate [101]. An aqueous dispersion containing a commercial anthocyanin extract (Chr. Hansen, Brazil) and guar gum, a galactomannan polysaccharide, at concentrations up to 1.75% at pH 4.0 improved the half-life of anthocyanins under thermal treatment (10 day-storage at 40 °C), with a 2.4-fold increase of *t*_1/2_ in the case of addition of 1.25% guar gum [102]. At higher concentration of guar gum (1.75%) the total content of anthocyanins decreased more than in the other cases, due to a higher viscosity, which restricts the anthocyanin molecules and consequently the H-bonding with the polysaccharide. The thermal stability of such complexes is explained by the H bonds formed between anthocyanin molecules and hydroxyl groups of guar gum. Similar experiments were performed by the same researchers using double emulsions W/O/W of anthocyanins, using guar gum (1.25%) and grape seed oil, confirming higher thermal stability of anthocyanins in the double emulsion associated with guar gum, for 10-day storage at 40 °C.

Nanocomposites of anthocyanins from black rice with silk fibroin peptide provided significant heat resistance of cyanidin-3-*O*-glucoside at 80 °C and tolerance to weakly acidic and alkaline conditions [103,104].

Blanching and processing the anthocyanin-containing products under controlled atmosphere (low oxygen) also have been used for improving thermal stability [88,105,106]. Blanching pre-treating of purple-fleshed sweet potato (*Ipomoea batatas* L.) by hot water for 1 min or steam for 1 min and atmospheric pressure, before hot air drying at 70 °C led to higher anthocyanin content than that of untreated samples [107]. Blanching inhibits the activity of some oxidative enzymes (peroxidase, lipoxygenase) responsible for the loss of anthocyanins. In the above-mentioned study, in addition, another strategy was applied to achieve both blanching and dehydration, namely microwave coupled to vacuum drying, confirming an improved anthocyanin thermal stability compared to that of samples subjected to blanching coupled to hot air drying.

**Figure 3:**
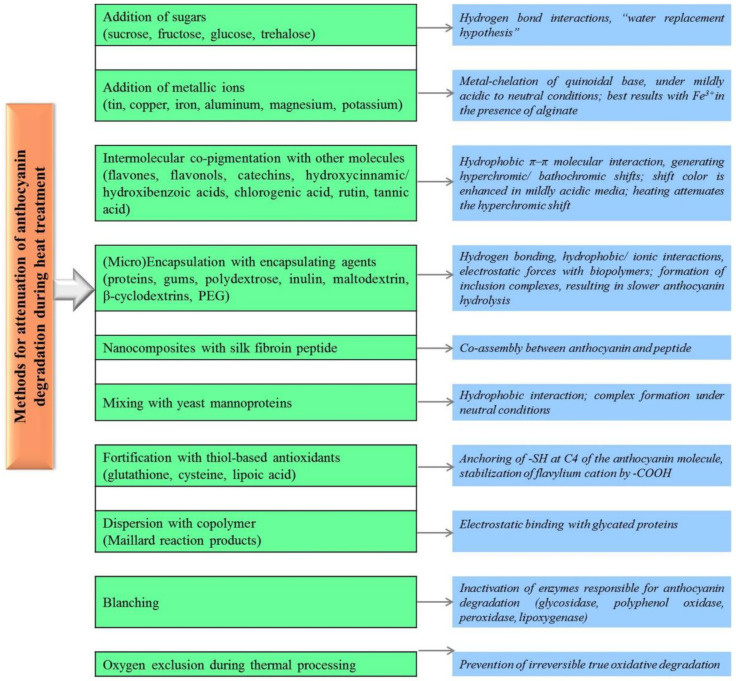
Schematic representation of useful methods for attenuation of the heat induced anthocyanin degradation and their proposed mechanism.

A brief summary of the most important and recent techniques applied for heat stabilization of anthocyanins and their mechanism of action is presented in Figure 3.

The results of a recently reported study regarding the heat stability of anthocyanins in extracts from fermented and unfermented grape skins after dehydration at 40 °C and 150 °C showed that fermentation may increase stability, fermented grape skins retaining more anthocyanin amounts and exhibiting higher antioxidant capacity as measured by DPPH assay, in particular at 40 °C, compared to unfermented fresh grape skins [108].

Progress on the thermal resistance of anthocyanins has been successfully achieved in the last few years by anchoring these molecules to inorganic matrices, e.g., mineral clays (saponite, palygorskite, sepiolite, montmorillonite), thus creating types of hybrid pigments to be used in composite colorimetric films to be applied for intelligent food packaging or monitoring food freshness [109,110,111]. The loading mechanism of natural pigments is based on the adsorption and intercalation of anthocyanins into the interlayer of inorganic matrix through electrostatic interaction and cation exchange [109]. The results of the TGA of hybrid powders of anthocyanins from *Lycium ruthenicum* fruits and montmorillonite showed an improved thermal stability of the natural pigments [110]. Moreover, synthetic analogues of anthocyanin chromophore (flavylium cations) or pyranoanthocyanins chromophore (pyranoflavylium cations) were successfully adsorbed on sepiolite clay to generate hybrid pigments stable in alkaline aqueous solution and resistant to thermal degradation [112]. The small molecules of synthetic flavylium cations adsorbed on sepiolite showed good color retention for 24 h at either 105 °C or 120 °C, compared to non-adsorbed control samples which degraded in less than 2 h at both temperatures and to pyranoflavylium cations adsorbed to sepiolite which degraded at higher temperatures. These findings are important for developing highly fluorescent hybrid pigments with enhanced color and thermal stability.

Controlling the critical parameters of conventional thermal processes of food remains a key operation to be deeply evaluated when heat-sensitive molecules are present in the matrix, until novel non-thermal technologies, such as pulsed electric field, pulsed light, ionizing and non-ionizing radiation, high-pressure processing/high hydrostatic pressure, cold plasma, ozone treatment, and ultrasounds [113,114] will be well understood and implemented in food systems.

## 5. Conclusions

Most food industrial processes require high temperature, mainly to ensure the safety of foodstuffs and extension of their shelf-life. Process temperature is a critical parameter that impacts on the food matrix, altering heat-sensitive compounds such as anthocyanins, in strong relation to magnitude and duration of heating.

The initial transformations of the chemical structure of anthocyanins under different pH and temperatures consist in reversible reactions of protonation, hydration and tautomery. Elevated temperatures shift the anthocyanin equilibria towards the tautomeric open-form, the colorless chalcone, which prevails in monoglycosidic anthocyanins, lacking a substituent at position C3. Glycosyl acylation of anthocyanins with organic acids usually enhance their heat stability, even in neutral media. At higher pH and temperature, anthocyanins are degraded to final derivatives of aldehyde and benzoic acid through irreversible oxidative reactions. Several kinetic models (Arrhenius, Eyring, Ball) have been applied to evaluate their thermal degradation. The mean value of the half-life of anthocyanin degradation at 60 °C is 19.7 h for fruits and 22.28 h for vegetables, and drastically decreased with increasing temperature, as deducted from several studies reviewed here. 

Crude extracts deliver more thermally stable anthocyanins than purified ones, probably due to inter- and intramolecular co-pigmentation reactions. In order to develop stable anthocyanin extracts/colorants in neutral media, the priority should be set at providing protection against autoxidation, for instance by the formation of stable redox-inert metal complexes or by adding suitable antioxidants. A different behavior pattern of anthocyanins within real food products subjected to thermal processing has been observed. Generally, thermal processing of simple food matrices appears to highly impact on anthocyanins, with their content loss varying from 28 to 80%. Thermal anthocyanin behavior could be different within more complex food matrices, e.g., in anthocyanin-enriched/fortified foods, because of the interactions with some nutrients (proteins, polysaccharides) which may stabilize these pigments.

Knowing the mechanism of anthocyanin degradation under various environmental conditions is essential for developing technological applications. Recently, innovative methods of anthocyanin stabilization under heating have enriched the data that already exist in the field. 

The scientific results reviewed here can help researchers and food manufacturers to understand the anthocyanin behavior under thermal processing and to apply the most suitable method for maximizing their retention and preserving their biological value.

## Figures and Tables

**Table 1:** Kinetic parameters, rate constant (*k*), half-time (*t*_½_) and activation energy (*E_a_*) of anthocyanin degradation under various heating conditions, in a selection of fruit, vegetable, flower and colorant products.

Source	pH	Heating *T* (°C)	Monitoring Time	Rate Constant (*k*)	*t*_½_ (h)	*E_a_* (kJ/mol)	Ref.
Fruits							
Açaí (*Euterpe precatoria* Mart.) pulp	nr	60	90 min	0.0005 min^−1^	23	42.8	[32]
		70		0.0006 min^−1^	19		
		80		0.0007 min^−1^	16		
		90		0.002 min^−1^	7		
Acerola (*Malpighia emarginata* D.C.) pulp juice	nr	60	1 h, 30 min	Graphical representation *k* = *f*(1/*T*)	4	68.042	[28]
		70			2		
		80			1		
		90			1/2		
Blackcurrants (*Ribes nigrum* L.) ethanol extract from thermally treated pulps	3.07	75	25 h	0.0065 h^−1^	21.9	94	[27]
		80		0.0131 h^−1^	7.2		
		90		0.0352 h^−1^	1.8		
Blueberries (*Vaccinium corymbosum* L.) ethanol extract from thermally treated pulps	3.46	70	25 h	0.0320 h^−1^	12.7	92	[27]
		80		0.0536 h^−1^	7.3		
		90		0.1167 h^−1^	1.4		
Blueberries/Rabbiteye (*Vaccinium achei*) juice	nr	50	25 h	0.273 × 10^−3^ min^−1^	42.30	80.42	[33]
		60		0.457 × 10^−3^ min^−1^	25.30		
		70	8 h	1.350 × 10^−3^ min^−1^	8.60		
		80		2.254 × 10^−3^ min^−1^	5.11		
Blueberries (*Vaccinium corymbosum* L.) methanol extract	3	50	10 h	0.009 h^−1^	74.47	nd	[34]
		60		0.026 h^−1^	26.51		
		70		0.047 h^−1^	14.73		
		80		0.146 h^−1^	4.73		
	6	50	10 h	0.044 h^−1^	15.64	nd	
		60		0.111 h^−1^	6.21		
		70		0.249 h^−1^	2.77		
		80		0.452 h^−1^	1.53		
Blackberries (*Rubus* spp.) acidified aqueous extracts	2	50	nr	1.8 × 10^−3^ min^−1^	6.4	15.0	[35]
		75		2.8 × 10^−3^ min^−1^	4.1		
		100		3.8 × 10^−3^ min^−1^	3.0		
Chokeberries (*Aronia* spp.) acidified aqueous extracts	2	50	nr	2.8 × 10^−3^ min^−1^	4.1	5.7	
		75		3.5 × 10^−3^ min^−1^	3.3		
		100		3.7 × 10^−3^ min^−1^	3.1		
Elderberries (*Sambucus* spp.) acidified aqueous extracts	2	50	nr	2.3 × 10^−3^ min^−1^	5.0	10.1	
		75		3.3 × 10^−3^ min^−1^	3.5		
		100		3.8 × 10^−3^ min^−1^	3.0		
Elderberry (*Sambucus nigra* L.) pigment isolates from concentrates	3.5	95	4 h	nr	1.96	nd	[11]
Grape (*Vitis vinifera* cv. of Karasakiz) juice	3.34	70	90 min	1.20 × 10^−3^ min^−1^	10.03	64.89	[29]
		80	60 min	2.40 × 10^−3^ min^−1^	5.02		
		90	60 min	4.20 × 10^−3^ min^−1^	2.79		
*Prunus nepalensis* L. (Sohiong), freeze dried extracts, in citrate phosphate buffer	3.5	50	7 h	0.018 days^−1^ (CE) 0.017 days^−1^ (EAE)	38.5 (CE) 40.7 (EAE)	nd	[36]
		80		0.044 days^−1^ (CE) 0.041 days^−1^ (EAE)	15.75 (CE) 16.9 (EAE)		
Strawberry (*Fragaria × ananassa* Duch.) pigment isolates from concentrates	3.5	95	4 h	nr	1.95	nd	[11]
Vegetables							
Black carrot (*Daucus carota* L. ssp. *sativus* var. *atrorubens* Alef.) pigment isolate from concentrate	3.5	95	4 h	nr	2.81	nd	[11]
Black rice (*Oryza sativa* L.) bran colorant powder dissolved in acetate buffer	3	60	2 h	0.71 × 10^−3^ min^−1^	16.3	45.05	[37]
		80		1.26 × 10^−3^ min^−1^	9.17		
		100		4.12 × 10^−3^ min^−1^	2.8		
	4	60		2.16 × 10^−3^ min^−1^	5.35	21.09	
		80		3.67 × 10^−3^ min^−1^	3.15		
		100		4.87 × 10^−3^ min^−1^	2.37		
	5	60		5.34 × 10^−3^ min^−1^	2.16	17.54	
		80		8.59 × 10^−3^ min^−1^	1.34		
		100		12.3 × 10^−3^ min^−1^	0.94		
Purple sweet potato (*Ipomoea batatas* L.), in citric buffer	3	70	6 h	236 × 10^−4^ h^−1^	29.4	16.46	[38]
		80		262 × 10^−4^ h^−1^	26.5		
		90		320 × 10^−4^ h^−1^	21.7		
Purple sweet potato (*Ipomoea batatas* L.) solution 5 cvs.: Mokpo No. 62, Borami, Jami, Sinjami and Ayamurasaki	3	60	-	0.04035, 0.03453, 0.03613, 0.03774 and 0.03800 h^−1^	17.2, 20.1, 19.2, 18.4 and 3.2 day	54.67, 60.93, 71.73, 59.35 and 62.28	[39]
		80		0.23364, 0.22338, 0.22222, 0.21935 and 0.21627 h^−1^	3.0, 3.1, 3.1, 3.2 and 3.2 day		
Red cabbage (*Brassica oleracea* L. var. *capitata* f. *rubra*) ethanol extract	3.5	80	7 h	1.7 × 10^−3^ min^−1^	6.7	nd	[40]
Red cabbage (*Brassica oleracea* L. var. *capitata* f. *rubra*) aqueous extract	nr	60	30 h	0.0273 h^−1^	25.3	nd	[41]
		70		0.0394 h^−1^	17.6		
		80		0.0694 h^−1^	10.0		
Flowers							
Hibiscus calyces (*Hibiscus sabdariffa* L.) 4 types of ethanol/methanol extracts acidified with HCl, formic acid, citric acid or acetic acid	3	70	6 h	0.0007, 0.0009, 0.0011 and 0.0009 min^−1^	22.0, 26.0, 18.0 and 19.0	nd	[42]
		75		0.0005, 0.0006, 0.0011 and 0.0007 min^−1^	18.0, 17.0, 12.0 and 17.0		
		80		0.0004, 0.0006, 0.0013 and 0.0007 min^−1^	19.0, 17.0, 10.0 and 13.0		
		85		0.0004, 0.0007, 0.0012 and 0.0005 min^−1^	18.0, 19.0, 16.0 and 16.0		
Purified anthocyanins							
Colorant—anthocyanins liquid (ColorFruit^®^ Violet 100 WS)	7	80	90 min	0.0114 min^−1^ (without stabilizer) 0.0027 min^−1^ (with mannoproteins)	50.4 min (without stabilizer) 272.4 min (with mannoproteins)	nd	[43]
		126		0.0271 min^−1^ (without stabilizer) 0.0051 min^−1^ (with mannoproteins)	25.8 min (without stabilizer) 143.4 (with mannoproteins)		
Cyanidin-3-*O*-glucoside	3	80	2 h	0.0018 min^−1^	386.3 min	nd	[44]
	6	80		0.0063 min^−1^	109.2 min		

Note: nr = not reported; nd = not determined; CE conventional extraction; EAE enzyme-aided extraction.

**Table 2:** Anthocyanin loss (%) in crude and purified extracts subjected to heating.

Source	Type of Extract	*T* (°C)	Conclusion on Anthocyanin Degradation	Ref.
Crude Extracts				
Black currant (*Ribes nigrum* cv. Ben Lomond)	Acidified ethanol extract (pH 4.1)	74	16.3% anthocyanin degradation with respect that of extraction conducted at 6 °C	[50]
Black grape pomace (*Vitis vinifera*)	Acidified ethanol extract (pH 3.0)	70	11.82% anthocyanin degradation after 120 min	[51]
		80	12.36% anthocyanin degradation after 120 min	
		90	49.79% anthocyanin degradation after 120 min	
Blueberry (*Vaccinium corymbosum* L.)	Methanol extract (pH 3.0)	50	Good anthocyanin preservation rate: 95% after heating at 50 °C for 10 h	[34]
		60	Anthocyanin preservation rate = 80% after heating at 60 °C for 10 h	
		80	Low anthocyanin preservation rate: 18.54% after heating at 80 °C for 10 h	
Elderberry (*Sambucus* *Nigra* L.)	Aqueous extract (pH 5.5)	70	~80% remaining absorbance at 535 nm after 3 h	[52]
		90	63.8% remaining absorbance at 535 nm after 6 h	
Black carrot (*Daucus carota* L.)	Acidified ethanol extract (pH 3.0)	70	9.36% anthocyanin degradation after 120 min	[51]
Red cabbage (*Brassica oleracea* L. var. *capitata f. rubra*)	Acidified ethanol extract/dried (pH 3.0 and pH 5.0)	70	2.57% anthocyanin degradation after 120 min (pH 3) 6.5% anthocyanin degradation after 120 min (pH 5)	[51]
		80	5.04% anthocyanin degradation after 120 min (pH 3) 24.64% anthocyanin degradation after 120 min (pH 5)	
		90	26.09% anthocyanin degradation after 120 min (pH 3) 33.33% anthocyanin degradation after 120 min (pH 5)	
	Aqueous extract (pH 5.5)	70	~70% remaining absorbance at 535 nm after 2 h	[52]
		90	46.1% remaining absorbance at 535 nm after 6 h	
Red onion outer skin (waste) (*Allium cepa* L.)	Ethanol extract; different acidic and alkaline conditions	Differential scanning calorimetry (DSC) method	The onset temperature, *T_on_*, of anthocyanin degradation was 51.74 °C under acidic conditions (pH = 4.5); the *T_on_* was 44.64 °C under alkaline conditions (pH = 9.0)	[53]
*Clitoria ternatea* (butterfly pea) blue petals	Aqueous extract (pH 3.6)	80	90% color retention at 617 nm	[54]
		90	76.1% color retention at 617 nm	
		100	70.2% color retention at 617 nm	
Hibiscus (*Hibiscus sabdariffa* L.)	Aqueous extract (pH 5.5)	50	More than 80% remaining absorbance at 535 nm after 2 h	[52]
		70	Less than 70% remaining absorbance at 535 nm after 2 h	
		90	26.7% remaining absorbance at 535 nm after 6 h	
Purified Extracts				
Black carrot (*Daucus carota* L.)	Purified anthocyanin powder extract (ColorFruit Carrot 12 WSP)	50	Kinetic parameters: *k* × 10^−2^ days = 0.92 *E_a_* = 63.2 kJ·mol^−1^	[55]
Purple potato (*Solanum tuberosum* cv. Purple Majesty)	Purified anthocyanins from acidified methanolic extract (pH 5.95)	100	Kinetic parameters, for 0–60 min: *t*_1/2_ = 26.456 min, *k* (min^−1^) × 10^3^ = 26.2, *E_a_* = 72.89 kJ·mol^−1^	[12]
		150	Kinetic parameters, for 0–60 min: *t*_1/2_ = 2.428 min, *k* (min^−1^) × 10^3^ = 285.5, *E_a_* = 72.89 kJ°mol^−1^	

**Table 3:** The fate of anthocyanins during thermal treatments of food containing these biomolecules.

Starting Raw Material	Final Product	Thermal Processing	Major Conclusions on Anthocyanins	Ref.
Blueberries (*Vaccinium corymbosum,* cv. Bluecrop)	Canned in syrup	Cans were exhausted for 4 min in a steam box at 87.8–93.3 °C; the sealed cans were immersed in boiling water for 15 min	28% anthocyanin loss	[59]
Pigmented potato (*Solanum tuberosum* L.) (Valfi, Blue Congo, Blaue St. Galler, Violette, Highland Burgundy Red)	Cooked	Cooking of whole tubers: (1) boiled water 15 min, (2) boiled steam 15 min, (3) microwave 9 min. Baking (40 min at 180 °C)	Anthocyanins increased by 4.2–4.5 times when boiled steam and boiled water was involved, and by 3.34 times by baking	[60]
Red cabbage (*Brassica oleracea* L. ssp. *capitata* f. rubra)	Cooked	Cooking (blanching, boiling, steaming)	59%, 41% and 29% respectively, loss in the anthocyanin content	[61]
Blackberries (*Rubus fruticosus* L.)	Jam	Boiling for 30 min Composition: 67% fruit, 33% sugar; no addition of pectin and citric acid	80% total anthocyanins degradation	[62]
Red raspberries (*Rubus idaeus* L.)	Jam	Boiling for 30 min Composition: 67% fruit, 33% sugar; no addition of pectin and citric acid	66% total anthocyanins degradation	[62]
Strawberries (*Fragaria x* *ananassa*, cultivars Chandler, Tudla and Oso Grande)	Jam	Thermal treatment for 15 min at 78 °C under vacuum, then heated at 92 °C; addition of pectin and citric acid to fruit composition	36–43% total anthocyanins degradation	[63]
Sweet cherries (*Prunus avium* L., cultivated and wild)	Jam	Boiling for 30 min Composition: 67% fruit, 33% sugar; no addition of pectin and citric acid	66% total anthocyanins degradation in cultivated cherries 80% total anthocyanins degradation in wild cherries	[62]
Blackberries (*Rubus* sp., cv. Apache	Juice	Blanching for 3 min at 95 °C, enzymatic treatment, pasteurization at 90 °C	~67% anthocyanin content decrease	[64]
Blueberries (*Vaccinium corymbosum* L.)	Juice	Thawing, depectinization at 43 °C, pasteurization at 90 °C for 1 min	32% of anthocyanins recovered in single-strength juice; 53% recovery of chlorogenic acid	[65]
Strawberries (*Fragaria ananassa* Duch, cultivar Camarosa)	Juice	Pasteurization: (a) 30 s at 90 °C (b) 60 s at 90 °C	4% anthocyanins degradation (30 s at 90 °C) 9% anthocyanins degradation (60 s at 90 °C)	[66]
Strawberries (*Fragaria ananassa*)	Juice	Pasteurization in glass bottles, for 15 min at 85 °C	21% anthocyanins degradation	[67]
Blueberries (*Vaccinium corymbosum*)	Dried fruits	Drying of whole fruits at 90 °C for 90 min, followed by 70 °C for 120 min, and finally 50 °C for 120 min	41% total anthocyanins degradation	[68]
Purple carrot (*Daucus carota* L., Deep Purple, Purple Haze)	Dried slices	Drying (1) convective, 70 °C; (2) microwave 40 °C; (3) freeze-drying	50% total anthocyanins degradation (convective drying) 20% total anthocyanins degradation (microwave/Deep Purple) 30% total anthocyanins degradation loss (freeze-drying/Purple Haze)	[69]
Purple potato (*Solanum tuberosum*)	Dried slices	Air-impingement jet drying of slices at 50, 65 and 80 °C	Kinetic parameters: *t*_1/2_ = 103.45 min (drying at 50 °C) *t*_1/2_ = 82.52 (drying at 65 °C) *t*_1/2_ = 64.78 (drying at 80 °C),	[70]
Strawberries (*Fragaria x* *Ananassa,* Dutch)	Dried slices	Drying of cut slices at 60, 70, 80 and 90 °C using a hot-air experimental tunnel dryer	Total anthocyanins degradation by: 37.04% (60 °C), 50.17% (70 °C), 52.91% (80 °C) 55.32% (90 °C)	[71]
Acerola (*Malpighia emarginata D.C.*)	Pulp	Industrial pasteurization (at 60, 70, 80 and 90 °C)	0.805% anthocyanin loss, at 90 °C for low residence time (20 s)	[28]
Blueberries (*Vaccinium corymbosum,* cv. Bluecrop)	Puree	Heating of blended berries at 95 °C, cooling and addition of corn syrup (18° Brix), heating at 92.8 °C, and canning in jars	43% anthocyanin loss	[59]
